# Age-Dependent Protein Aggregation Initiates Amyloid-β Aggregation

**DOI:** 10.3389/fnagi.2017.00138

**Published:** 2017-05-17

**Authors:** Nicole Groh, Anika Bühler, Chaolie Huang, Ka Wan Li, Pim van Nierop, August B. Smit, Marcus Fändrich, Frank Baumann, Della C. David

**Affiliations:** ^1^Protein Aggregation and Aging, German Center for Neurodegenerative DiseasesTübingen, Germany; ^2^Graduate School of Cellular and Molecular NeuroscienceTübingen, Germany; ^3^Hertie Institute for Clinical Brain Research, Department of Cellular NeurologyTübingen, Germany; ^4^Department of Molecular and Cellular Neurobiology, Center for Neurogenomics and Cognitive Research, Amsterdam Neuroscience, VU University AmsterdamAmsterdam, Netherlands; ^5^Institute of Protein Biochemistry, Ulm UniversityUlm, Germany

**Keywords:** amyloid-β, protein aggregation, aging neuroscience, heterologous seeding, *C. elegans* aging, mouse brain

## Abstract

Aging is the most important risk factor for neurodegenerative diseases associated with pathological protein aggregation such as Alzheimer’s disease. Although aging is an important player, it remains unknown which molecular changes are relevant for disease initiation. Recently, it has become apparent that widespread protein aggregation is a common feature of aging. Indeed, several studies demonstrate that 100s of proteins become highly insoluble with age, in the absence of obvious disease processes. Yet it remains unclear how these misfolded proteins aggregating with age affect neurodegenerative diseases. Importantly, several of these aggregation-prone proteins are found as minor components in disease-associated hallmark aggregates such as amyloid-β plaques or neurofibrillary tangles. This co-localization raises the possibility that age-dependent protein aggregation directly contributes to pathological aggregation. Here, we show for the first time that highly insoluble proteins from aged *Caenorhabditis elegans* or aged mouse brains, but not from young individuals, can initiate amyloid-β aggregation *in vitro*. We tested the seeding potential at four different ages across the adult lifespan of *C. elegans*. Significantly, protein aggregates formed during the early stages of aging did not act as seeds for amyloid-β aggregation. Instead, we found that changes in protein aggregation occurring during middle-age initiated amyloid-β aggregation. Mass spectrometry analysis revealed several late-aggregating proteins that were previously identified as minor components of amyloid-β plaques and neurofibrillary tangles such as 14-3-3, Ubiquitin-like modifier-activating enzyme 1 and Lamin A/C, highlighting these as strong candidates for cross-seeding. Overall, we demonstrate that widespread protein misfolding and aggregation with age could be critical for the initiation of pathogenesis, and thus should be targeted by therapeutic strategies to alleviate neurodegenerative diseases.

## Introduction

A variety of neurodegenerative diseases are associated with the misfolding and aggregation of specific proteins. In Alzheimer’s disease (AD), amyloid-β (Aβ) peptides and tau proteins aggregate and ultimately form the characteristic pathological hallmarks: amyloid plaques and neurofibrillary tangles (NTFs) respectively. In recent years, understanding the initiation and spread of these hallmark protein aggregates has become a central area of investigation (Jucker and Walker, 2011). The current model stipulates that aggregation in disease is initiated by a protein seed that forms a template for further protein aggregation (Jucker and Walker, 2013). Support for this model comes from research showing that the exogenous addition of minute amounts of Aβ or tau seeds greatly accelerates the onset of aggregation both *in vitro* and *in vivo* (Clavaguera et al., 2009; Langer et al., 2011; Nagarathinam et al., 2013). An important and currently understudied question is how aging influences protein aggregation in neurodegeneration. Recently, physiological protein insolubility in the context of aging has become a hot topic of research (Partridge, 2011; David, 2012). Indeed, numerous publications demonstrate that protein aggregation is not restricted to disease but a normal consequence and possibly cause of aging (David et al., 2010; Demontis and Perrimon, 2010; Peters et al., 2012; Reis-Rodrigues et al., 2012; Ottis et al., 2013; Walther et al., 2015; Ayyadevara et al., 2016b; Tanase et al., 2016; Lechler et al., 2017).

Until now, it remains unclear whether and how age-dependent protein aggregation and disease-associated protein aggregation influence each other. One possibility is that age-dependent aggregates indirectly accelerate disease-associated protein aggregation by stressing the cell and/or titrating away anti-aggregation factors. Another possibility is a direct interaction whereby disease-associated proteins and age-dependent aggregation-prone proteins co-aggregate. In support of this latter hypothesis, proteins prone to aggregate during normal aging are significantly overrepresented as minor protein components in amyloid plaques and NFTs (David et al., 2010; Ayyadevara et al., 2016a). Recent research reveals that the sequestration of these age-dependent aggregation-prone proteins in the disease aggregates is a source of toxicity (Ayyadevara et al., 2016a). However, whether misfolded proteins aggregating with age can form heterologous seeds that initiate Aβ aggregation (see model Supplementary Figure 1A) has not been investigated. Although current research focuses on homologous seeding, there are a few examples of cross-seeding (or heterologous seeding) mostly between different disease-aggregating proteins (Morales et al., 2013). For instance, Aβ is a potent seed for the aggregation of human islet amyloid polypeptide (hIAPP) involved in type II diabetes (O’Nuallain et al., 2004; Oskarsson et al., 2015); Aβ and prion protein PrP^Sc^ cross-seed each other and accelerate neuropathology (Morales et al., 2010); and both α-synuclein and Aβ co-aggregate with tau and enhance tau pathology *in vivo* (Guo et al., 2013; Vasconcelos et al., 2016). Finally, we recently showed that cross-seeding between different age-dependent aggregating proteins is possible in the absence of disease (Lechler et al., 2017).

Here, we demonstrate that cross-seeding during aging is likely to be an important mechanism underlying protein aggregation in AD.

## Materials and Methods

### *C. elegans* Mutants

CF2253: *gon-2(q388)I.*

### *C. elegans* Transgenics

GMC101: dvIs100[unc-54p::A-beta-1–42::unc-54 3′-UTR + mtl-2p::GFP]
UE50: oaSi10[par-5p::GFP::par-5::par-5 3′-UTR + unc-119(+)]
DCD296 (GMC101;UE50): dvIs100[unc-54p::A-beta-1–42::unc-54 3′-UTR + mtl-2p::GFP]; oaSi10[par-5p::GFP::par-5::par-5 3′-UTR + unc-119(+)].

### Mouse Strains

Wild-type C57BL/6J (WT) and transgenic APP23 mice (Sturchler-Pierrat et al., 1997) were bred and maintained under pathogen-free conditions at the Hertie Institute for Clinical Brain Research. All studies were performed in accordance with German animal welfare legislation and with approval from the Ethical Commission for animal experimentation of Tübingen, Germany. Age and sex of mice used are listed in Supplementary Table 1.

### *C. elegans* Culture

To obtain large aged-synchronized populations of *Caenorhabditis elegans*, temperature induced sterile *gon-2* mutants were used. Eggs and L1s were collected from adults grown at 20°C. After L1 arrest overnight at 20°C, worms were grown until L4 stage and shifted to 25°C. Then eggs and L1s were collected from these 1-day-old adults. After an arrest overnight at 25°C, L1s were counted and divided into four liquid cultures (to obtain day 2, day 6, day 10, and day 14 time points) and grown with OP50-1 (OP50 with Streptomycin resistance) at 25°C. The whole procedure was carried out independently four times to collect four biological replicates. The total numbers of worms grown in the four liquid cultures for each replicate are shown in Supplementary Table 2. At day 2, day 6, day 10, and day 14 worms were collected and removed from bacteria and dead worms by sucrose separation as previously described (David et al., 2010). Age is defined by the number of days of adulthood starting from the last larval stage L4. The resulting worm pellets were frozen in liquid nitrogen with an equal volume of RAB high-salt (0.1 M MES, 1 mM EGTA, 0.1 mM EDTA, 0.5 mM MgSO_4_, 0.75 M NaCl, 0.02 M NaF, 1 mM PMSF, Roche Complete Inhibitors 2x). Frozen worm pellets of each day were ground in a mortar.

To obtain a large population of the *C. elegans* transgenic strain GMC101 which overexpresses Aβ as positive control for the FRANK assay (Fibrillization of Recombinant Aβ Nucleation Kinetic), we used the same protocol as described above with the following changes: after an L1 arrest overnight at 20°C 150,000 worms were grown in liquid culture at 20°C until day 1 and shifted to 25°C to induce paralysis (McColl et al., 2012). At day 2 worms were collected as described.

### Insoluble Protein Extraction with *C. elegans*

To isolate SDS-insoluble proteins for mass spectrometry analysis and the FRANK assay, we performed a sequential extraction as previously described (David et al., 2010). For mass spectrometry analysis, 350 mg ground worms per time point were solubilized in two volumes of high-salt RAB (0.1 M MES, 1 mM EGTA, 0.1 mM EDTA, 0.5 mM MgSO_4_, 0.75 M NaCl, 0.02 M NaF, 1 mM PMSF, Roche Complete Inhibitors 2x, 200 U/ml DNaseI and 100 μg/ml RNaseA). For the FRANK assay, 50 mg ground worms (*gon-2* mutants or Aβ overexpressing transgenics) per time point were solubilized in 200 μl RAB. High-salt soluble proteins were removed by centrifugation at 18,400 *g* for 20 min at 4°C. After dialysis against PBS, these high-salt soluble proteins of *gon-2* mutants were used as soluble fraction in the FRANK assay. The pellet was reextracted in RAB with 1 M sucrose to help remove lipids and then resuspended twice in two volumes of RIPA buffer (respectively 200 μl for the FRANK assay extraction) (50 mM Tris pH 8, 150 mM NaCl, 5 mM EDTA, 0.5% SDS, 0.5% SDO, 1% NP-40, 1 mM PMSF, Roche Complete Inhibitors 1×). Detergent soluble proteins were removed by centrifugation at 18,400 *g* for 20 min at 4°C. This final pellet containing highly insoluble proteins was resuspended in 400 μl 70% formic acid for mass spectrometry and centrifuged at 50,000 *g* for 20 min at 4°C, to remove worm cuticle debris. For the FRANK assay, the final pellet was resuspended in 100 μl PBS and centrifuged at 3,000 *g* for 5 min at 4°C to remove the worm cuticle.

To isolate insoluble proteins for western blot analysis, a simplified protocol was used where 50 mg of ground animals in RAB buffer were directly resuspended in 150 μl RIPA buffer. SDS-insoluble proteins were isolated by washing with RIPA buffer and centrifugation at 18,400 *g* for 20 min at 4°C. The pellet was washed once in 100 μl RIPA. Final pellets containing insoluble proteins were recovered in 75 μl 8 M Urea, 2% SDS, 50 mM DTT, 50 mM Tris pH 8 at room temperature.

### Mouse Brain Preparation and Insoluble Protein Extraction for the FRANK Assay

Brains from wild-type mice (Supplementary Table 1) were divided into both hemispheres after the cerebellum had been removed. The same was done with the mouse brain from a transgenic APP23 mouse (20 months). Two volumes per weight high-salt RAB (see above) were added per hemisphere. Each hemisphere was homogenized with the Precellys Ceramic Beads Kit (Cayman Chemical). During each extraction step, two volumes buffer per weight were used. High-salt soluble proteins were removed by centrifugation at 18,400 *g* for 20 min at 4°C. Insoluble proteins were isolated as was performed with *C. elegans*. The resulting pellet after the last RIPA step was resuspended in 200 μl PBS. The sample was centrifuged at 3,000 *g* for 5 min at 4°C and the supernatant containing the detergent-insoluble proteins was used for the FRANK assay.

### FRANK Assay (Fibrillization of Recombinant Aβ Nucleation Kinetic)

The seeding potential of soluble or insoluble protein extracts from *C. elegans* and from wild-type mouse brains was detected by measuring Thioflavin T fibrillization kinetics as described by Nagarathinam et al. (2013) with the modifications as described in detail in Marzesco et al. (2016). In brief: all kinetic measurements were carried out with 20 μM soluble recombinant Aβ(1–40) in 50 mM Phosphate buffer pH 7.4 and 150 mM NaCl with 20 μM ThT supplemented with protease inhibitors (Roche Complete Inhibitors). Each assay was performed with freshly monomerized Aβ1–40. Briefly, lyophilized recombinant Aβ1–40 peptide was dissolved to a stock-concentration of 5 mM in 100% DMSO and frozen at -80°C. Before use, this stock was then freshly diluted in DMSO to 400 μM and sonified for 20 min in a water bath followed by 30 min centrifugation at 22,000 *g* at room temperature. The supernatant was then further diluted to reach 200 μM Aβ1–40 in a 50% DMSO stock. In 96-well plates (μclear non-bind plate; Greiner), eight technical replicates were measured for each sample. The plates sealed with film sheets were incubated at 37°C for up to 2–3 days. The fluorescence measurements were performed from the bottom of the plate on a Fluostar Omega plate reader (BMG Labtech) (excitation: 440 nm, emission: 480 nm) at 30-min interval after double orbital shaking for 30 s at 500 rpm. For each sample, five to eight replicates were averaged and the lag times were determined from fitted curves (Nielsen et al., 2001) with GraphPad Prism 5. For this assay, the peptide Aβ(1–40) was expressed and purified as described earlier (Hortschansky et al., 2005). Bicinchoninic acid (BCA) protein assay was performed to determine the total protein content in the soluble or detergent-insoluble protein fractions. For all experiments (with *C. elegans* and mice) except for Supplementary Figure 2, we used 0.001 μg.

### Paralysis Assay

To compare the paralysis levels of worms overexpressing PAR-5 (UE50), Aβ (GMC101) or both (double transgenic, DCD296), 105 L4s of each overexpressing strain were kept at 20°C. At day 1 of adulthood, worms were transferred to 25°C to induce paralysis as previously described (McColl et al., 2012). The numbers of paralyzed worms were counted from day 2 to day 4 (until most of the double-transgenic worms were paralyzed).

### Statistics

**FIGURE 1:**
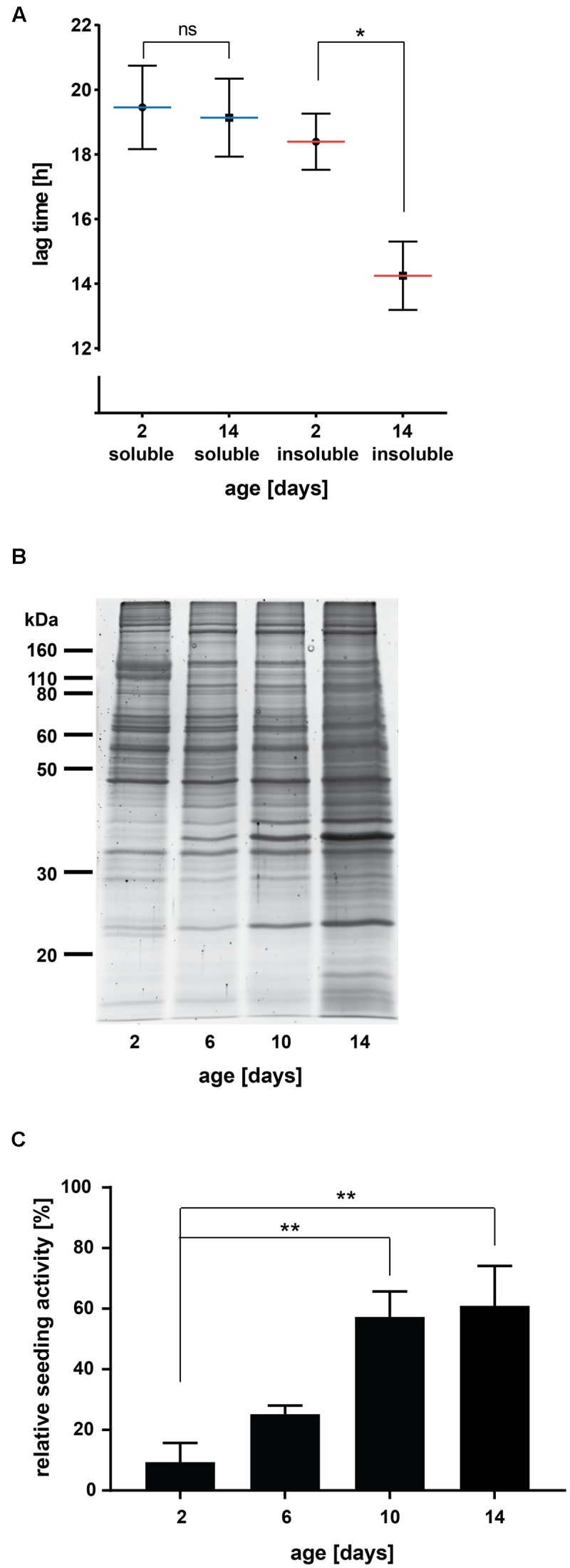
**Insoluble protein extracts from aged *C. elegans* seed Aβ aggregation *in vitro*. (A)** Lag times measured for Aβ aggregation in the presence of 0.001 μg *C. elegans* soluble and detergent-insoluble protein extracts (day 2 and day 14). Mean values (blue lines soluble, red lines insoluble) with respective SEM of three biological replicates are represented (each including a minimum of five technical replicates). Two-tailed *p*-value: day 2 insoluble vs. day 14 insoluble ^∗^*p* = 0.039. **(B)** SDS gel of insoluble protein extracts from different ages stained with Sypro Ruby protein gel stain. **(C)** Relative seeding activity measured for Aβ aggregation in the presence of 0.001 μg insoluble protein extracts from different ages. Mean values of four biological replicates (each including five technical replicates) are shown. Data were normalized with 0% being the lag time of the negative control (soluble extract, day 2) and 100% being the lag time of the positive control (insoluble extract from *C. elegans* transgenics expressing Aβ). One-way ANOVA: day 2 vs. day 10 ^∗∗^*p* = 0.0086 and day 2 vs. day 14 ^∗∗^*p* = 0.0051.

For the FRANK Assays significance was tested with unpaired *t*-test (*p* < 0.05), one-way ANOVA (*p* < 0.05) or two-way ANOVA (*p* < 0.05) using Graph Pad Prism 7. For **Figure 1C** the relative seeding activity was calculated using the normalization function of Graph Pad Prism 7. The absolute lag times measured for four biological replicates were normalized to those of the standards. The lag time of a soluble extract from day 2 worms (negative control) was defined as 0% and the lag time of an insoluble extract from transgenic *C. elegans* overexpressing Aβ (GMC101, positive control) was defined as 100%.

For the comparison of the paralysis levels of PAR-5 overexpressing worms, Aβ overexpressing worms and the double-transgenic worms overexpressing both proteins, Fisher’s exact test was used^1^.

### Mass Spectrometry Analysis

Sodium dodecyl sulfate-insoluble proteins dissolved in formic acid were further processed for mass spectrometry analysis as previously described (David et al., 2010). Briefly, after dialysis, proteins were solubilized in a final concentration of 8 M Urea. After alkylation and reduction, samples were diluted in 150 mM ammonium bicarbonate to obtain a final 2 M Urea concentration. Proteins were digested by modified trypsin (Promega) at 5% w/w overnight at 37°C. Peptides from *C. elegans* at day 2, day 6, day 10, and day 14 from two biological replicates were labeled by 8-plex iTRAQ following the manufacturer’s instructions (Applied Biosystems).

Mass spectrometry and data analysis were performed as described (Klemmer et al., 2011). The iTRAQ-tagged samples were pooled, dried and peptides partially separated in a strong cation exchange column (2.1 mm × 150-mm PolySUFOETHYL A column, PolyLC, Inc.). Peptides in each collected fractions were further fractionated by capillary reverse phase C18 column (150 mm × 100 μm-inner diameter column packed in house with the Alltima C18 3 μm particle using the pressure injection cell from Next Advance). The eluents were continuously mixed with matrix (α-cyanohydroxycinnamic acid), and deposited off-line to the metal target every 15 s. The peptides were analyzed on the 5800 proteomics analyzer (AB-Sciex). MS/MS spectra were each collected from 2500 laser shots; a maximum of 25 MS/MS was allowed per spot. Mascot was used to annotate spectra that were searched against *C. elegans* UniprotKB database.

The peak areas of each iTRAQ signature ions were log2-transformed and normalized to the total peak area of the signature peaks. The peak areas in each sample were mean-centered, which were used to calculate the protein averages. The permutation-derived false discovery rate (*q*-value) were calculated by the excel plug-in of the significant analysis of microarrays (SAM) program (Van Nierop and Loos, 2011).

### Ingenuity Pathway Analysis

Human homologs of early-aggregating proteins and late-aggregating proteins in *C. elegans* (as described in the results) were identified using cildb 3.0^2^ and Ensembl genome browser 87^3^. 83 human homologs of early-aggregating proteins and 116 human homologs of late-aggregating proteins including early-aggregating proteins that continued to aggregate strongly at day 10 and day 14 were analyzed together with 161 minor components of AD pathological aggregates previously published (Liao et al., 2004; Wang et al., 2005; Ayyadevara et al., 2016a) using Ingenuity Pathway Analysis (IPA; Winter Release 2016^4^). Fisher’s exact test was used to calculate a *p*-value reflecting the probability that the association between the set of molecules and a given pathway is due to chance alone. Our threshold of the *p*-value for association was set to 0.01.

### Western Blot

Nine μl Urea/SDS samples (insoluble proteins) were loaded on a 4–12% gradient gel. The membrane was probed with anti-14-3-3 (1:5000, SC-1657, Santa Cruz Biotechnology). The quantification was done with ImageJ.

## Results

### Protein Aggregates Formed during Normal *C. elegans* Aging Seed Aβ Aggregation *In Vitro*

We first investigated whether age-dependent protein aggregates formed during aging in *C. elegans* have the potential to seed the aggregation of synthetic Aβ *in vitro*. *C. elegans* has been extensively characterized as a model for aging (Antebi, 2007) and widespread protein aggregation with age has been repeatedly documented (David et al., 2010; Reis-Rodrigues et al., 2012; Walther et al., 2015). We isolated highly detergent-insoluble proteins from young and aged *C. elegans* as previously described (David et al., 2010). As extensive protein aggregation occurs in the reproductive tissues (David et al., 2010), we used a gonad-less *C. elegans* strain to investigate only somatic age-dependent protein aggregation. To identify seeding, we examined changes in the lag time preceding the formation of Aβ(1–40) fibrils *in vitro* using the FRANK assay (Fibrillisation of Recombinant Aβ Nucleation Kinetic) (Supplementary Figure 1B). This assay has been successfully used to quantify the seeding activity of different cellular extracts containing Aβ seeds (Nagarathinam et al., 2013; Fritschi et al., 2014; Marzesco et al., 2016).

We found that insoluble protein extracts from aged *C. elegans* (day 14, ∼50% dead) significantly shortened the lag time of Aβ aggregation compared to extracts from young animals (day 2, 0% dead) (**Figure 1A**, *p* **=** 0.039). Similar to a classical western blot, it is only meaningful to compare absolute lag time values evaluated in the same assay plate. However, despite variations between experiments, the difference in seeding activity between insoluble extracts from young and aged animals was highly reproducible (**Figure 1** and Supplementary Figure 2). Consistent with the nature of a seed, we found that only 0.001 μg of insoluble proteins from aged animals were needed to seed Aβ aggregation (Supplementary Figure 2). With higher amounts of insoluble proteins (0.01 or 0.1 μg), we observed a shorter lag time for Aβ aggregation also in the samples from young animals. This age-independent effect was eliminated by diluting the samples, whereas the seeding effect of insoluble samples from aged individuals remained unchanged. It is possible that this small seeding effect in young extracts was due to some contaminants or low concentrations of insoluble proteins undetectable after dilution.

To distinguish whether seeding is a general characteristic of the aged proteome or specific to aggregated proteins, we evaluated high-salt soluble proteins with the *in vitro* assay. The lag time for Aβ aggregation remained similar for soluble proteins from aged and young animals and comparable to that observed with insoluble extracts from young animals (**Figure 1A**). Together, these results demonstrate that protein aggregates appearing during normal aging can serve as heterologous seeds for Aβ aggregation.

### Seeding Activity Appears in the Later Stages of Life

Sporadic AD is a late-onset disease affecting humans over age 65. However, it remains unclear when pathogenesis starts in the patients’ brains. We analyzed insoluble extracts from different stages of adulthood to determine when the seeds triggering Aβ aggregation appear. For this, we chose four time points: young animals (day 2), early middle-aged animals (day 6, at the end of reproduction in wild-type animals), late middle-aged animals (day 10, ∼30% of the population has died) and old animals (day 14, ∼50% of the population has died). To collect large numbers of individuals for all time points, we cultured *C. elegans* in liquid. The whole procedure was performed four times to obtain four different biological replicates. In order to compare the seeding activities of all insoluble extracts measured in two separate assay plates, we included two standard extracts: a soluble extract as negative control and an insoluble extract from transgenic *C. elegans* overexpressing Aβ as positive control. The relative seeding activity was calculated by normalizing the absolute lag times measured for each biological replicate to those of the standards with 0% being the lag time of the negative control and 100% being the lag time of the positive control. Sypro Ruby staining of insoluble proteins on an SDS gel showed accelerated protein aggregation with increasing age, especially between day 10 and day 14 (**Figure 1B**). Evaluation of the relative seeding activity did not reveal a significant increase in seeding between day 2 and day 6 (**Figure 1C**, *p* = 0.57). Instead, we found a large increase in seeding activity at day 10 (*p* = 0.0086 compared to day 2). The seeding potential was not further increased in insoluble extracts from 14-day-old animals. When extrapolated to human aging, the appearance of heterologous protein aggregate seeds for Aβ aggregation at the later stages of life rather than early middle age would be consistent with the late-onset of AD dementia.

### Identification of Early- and Late-Aggregating Proteins during Aging

**FIGURE 2:**
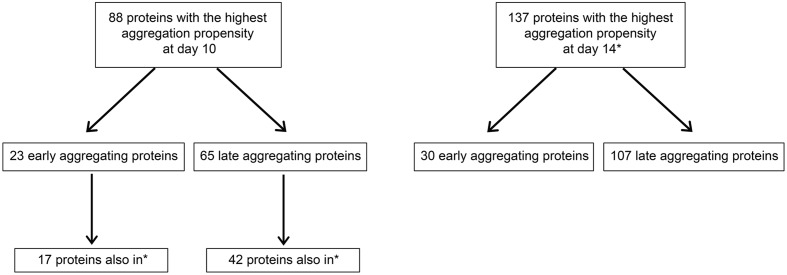
**Schematic summarizing numbers of early- and late-aggregating *C. elegans* proteins detected by quantitative mass spectrometry (also see Supplementary Tables 3**, **4**). Proteins with the highest aggregation propensity at day 10 or day 14 are defined as proteins quantified in the top 25th percentile compared to day 6. Proteins with the highest aggregation propensity at day 6 are defined as proteins quantified in the top 25th percentile compared to day 2. Quantification was performed with two biological replicates and all numbers represent only proteins in the top 25th percentiles from both replicates. ^∗^Proteins also among the 137 proteins with the highest aggregation propensity at day 14 (in top right box).

The results from the *in vitro* assay imply that changes in aggregation between day 6 and day 10 are responsible for seeding. We performed quantitative mass spectrometry using the stable-isotope iTRAQ reagents to identify these changes with two independent biological repeats. We identified 845 quantifiable aggregation-prone proteins (Supplementary Table 3). Of these, 460 were identified in our previous study (David et al., 2010). This large overlap attests to the quality of the present mass spectrometry analysis. After normalization, we ranked the proteins depending on their relative change in aggregation levels and we focused on proteins with the highest change in aggregation with age, i.e., in the top 25th percentile in both repeats. Early-aggregating proteins were defined as present in the top 25th percentile of day 6 compared to day 2 and late-aggregating proteins as present in the top 25th percentile of day 10 or day 14 compared to day 6 (**Figure 2**). We detected 133 proteins at day 6 in the top aggregating fractions in both replicates. Significantly among these early-aggregating proteins, the rate of aggregation continued to increase strongly for 23 proteins at day 10 (in the top 25th percentile) as well as 30 proteins at day 14 (**Figure 2**; as opposed to 3.3 by chance, Supplementary Table 4). Consequently, these latter proteins could also be responsible for seeding Aβ. At day 10 and day 14, we detected 65 and 107 late-aggregating proteins, respectively. Of these, 42 proteins (as opposed to 3.3 by chance, Supplementary Table 4) were prone to aggregate strongly at both later ages compared to day 6. As seeding activity appears in day 10 animals, these late-aggregating proteins are prime candidates.

To identify functional groups associated with the early- and late-aggregating proteins, we performed an IPA with the human homologs. Then, we investigated which of these pathways were also significantly associated with the minor components in AD pathological aggregates. This analysis highlighted eight pathways that tend to harbor both late-aggregating proteins and disease-related aggregate components, namely: proteins related to 14-3-3 mediated signaling, PI3K/AKT signaling, ERK/MAPK signaling, Calcium Transport I as well as proteins related to the ubiquitination pathway, aryl hydrocarbon receptor signaling, NRF2-mediated oxidative stress response and xenobiotic metabolism signaling (**Table 1** and Supplementary Table 5). The latter four categories were also significantly associated with early-aggregating proteins. Our present results predict that interactions between Aβ or tau with these pathway components could potentially induce or accelerate their aggregation.

**Table 1:** Ingenuity canonical pathways identified in the set of late- or early-aggregating proteins and their association with minor components found in AD pathological aggregates [–log(*p*-value) > 2].

	-log(*p*-value)	
Ingenuity canonical pathways	Late-aggregating proteins^∗^	Minor components
Protein ubiquitination pathway	6.82	2.57
Aryl hydrocarbon receptor signaling	4.93	3.19
Glutathione-mediated detoxification	4.75	
EIF2 signaling	4.54	
tRNA charging	4.23	
mTOR signaling	3.96	
LPS/IL-1 mediated inhibition of RXR function	3.67	
Regulation of eIF4 and p70S6K signaling	3.66	
PI3K/AKT signaling	3.23	5.37
ERK/MAPK signaling	3.11	3.18
Citrulline biosynthesis	3.09	
Calcium transport I	2.89	7.47
Death receptor signaling	2.79	
Superpathway of citrulline metabolism	2.59	
NRF2-mediated oxidative stress response	2.38	4.96
Xenobiotic metabolism signaling	2.31	2.92
14-3-3-mediated signaling	2.25	13.4
Endoplasmic reticulum stress pathway	2.24	
Glutathione redox reactions I	2.20	
Lipid antigen presentation by CD1	2.06	
	
	**Early-aggregating proteins**	**Minor components**
	
EIF2 signaling	5.68	
Protein ubiquitination pathway	5.22	2.57
Cell cycle control of chromosomal replication	4.87	
Aryl hydrocarbon receptor signaling	4.78	3.19
NRF2-mediated oxidative stress response	4.01	4.96
Creatine-phosphate biosynthesis	3.84	
Glutathione-mediated detoxification	3.73	
Leucine degradation I	3.29	
Xenobiotic metabolism signaling	3.08	2.92
Unfolded protein response	2.93	
eNOS signaling	2.52	
Aldosterone signaling in epithelial cells	2.42	2.05
Glutamine biosynthesis I	2.42	
Prostate cancer signaling	2.25	
Choline degradation I	2.12	

*^∗^Including early-aggregating proteins that continued to aggregate strongly at day 10 and day 14*.

**FIGURE 3:**
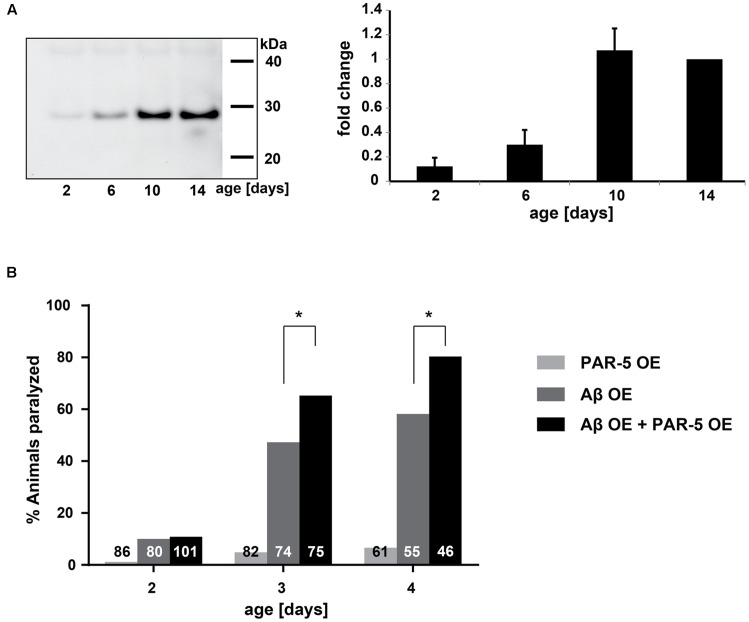
**Late-aggregation-prone protein PAR-5 accelerates Aβ toxicity in *C. elegans*. (A)** Left: representative immunoblot detecting PAR-5 (14-3-3) in insoluble protein extracts from different ages. Right: immunoblot quantification of PAR-5. Band intensities were normalized to day 14. *N* = 4 (biological repeats), SEM depicted. **(B)** Paralysis levels of worms that overexpress PAR-5 (PAR-5 OE), Aβ (Aβ OE) or both (double transgenic, Aβ OE + PAR-5 OE). Shown are the percentages of worms paralyzed at different days. The numbers in the bars represent the total numbers of worms analyzed. Fisher’s exact test: Aβ OE vs. Aβ OE + PAR-5 OE, day 3 ^∗^*p* = 0.032 and day 4 ^∗^*p* = 0.019.

In addition to the pathway analysis, we identified specifically which late-aggregating proteins were also minor components in AD. At day 10 and/or day 14 the homologs of the following 10 proteins were identified in amyloid plaques and/or NFTs: PAR-5, UBA-1, SPC-1, LMN-1, NEX-3, HIS-1, NKB-3, FRM-1, MCA-3, and GPD-1 (**Table 2**). Conversely, six minor components of amyloid plaques and/or NFTs were among the early-aggregating proteins in *C. elegans*. Of note, only one of these early-aggregating minor components, GST-1, continued to aggregate strongly at day 10 and day 14 and therefore could also play a role in Aβ seeding. Importantly, seeding activity of the late-aggregating protein, 14-3-3 (human homolog of PAR-5), has already been demonstrated both *in vitro* and in cell culture, whereby 14-3-3 initiates tau fibril formation (Hernandez et al., 2004; Qureshi et al., 2013; Li and Paudel, 2016). By western blot analysis, we confirmed that PAR-5 was highly prone to aggregate at day 10 compared to day 6 (3.2 fold, **Figure 3A**). In *C. elegans*, Aβ toxicity in the body-wall muscle causes the animals to paralyze (Link, 1995; McColl et al., 2012). We speculated that overexpression of the aggregation-prone protein PAR-5 would accelerate the rate of paralysis induced by Aβ. Indeed, we observed a significant increase in the number of double transgenics paralyzed compared to those solely expressing Aβ (**Figure 3B** and Supplementary Figure 3). This effect cannot be explained by a general toxicity due to PAR-5 over-expression as these animals displayed negligible levels of paralysis in the absence of Aβ.

**Table 2:** Minor components of AD pathological aggregates identified as late- or early-aggregating proteins in *C. elegans*.

*C. elegans* entry name	*C. elegans* Uniprot ID	*C. elegans* gene name	*Homo sapiens* protein name (homologs)
**Late-aggregating proteins**			
14331_CAEEL^x,†^	P41932	par-5	14-3-3 proteins ß/α; δ
C1P636_CAEEL^x^, ^∗^	C1P636	uba-1	Ubiquitin-like modifier-activating enzyme 1
G4S034_CAEEL^x^, ^∗^	G4S034	spc-1	Spectrin α-chain, non-erythrocytic protein 1
LMN1_CAEEL^x^, ^∗^	Q21443	lmn-1	Lamin A/C
Q27473_CAEEL^‡^, ^∗^	Q27473	nex-3	Annexin A5
H4_CAEEL^‡^	P62784	his-1	H4 histone family, member C
AT1B3_CAEEL^‡^	Q9XUY5	nkb-3	Sodium/potassium-transporting ATPase beta-1 chain
G5EEG8_CAEEL^x^	G5EEG8	frm-1	Band 4.1-like protein 1
Q95XP6_CAEEL^x^	Q95XP6	mca-3	Plasma membrane Ca^++^-transporter ATPase 1
G3P1_CAEEL^‡^	P04970	gpd-1	Glyceraldehyde-3-phosphate dehydrogenase
**Early-aggregating proteins**			
GSTP1_CAEEL^x,+^	P10299	gst-1	Glutathione *S*-transferases Mu 3, Mu 5
DYHC_CAEEL^x,†^	Q19020	dhc-1	Cytoplasmic dynein 1 heavy chain 1
HSP90_CAEEL^x,†^	Q18688	daf-21	Protein HSP90-β
G5ECP9_CAEEL^x^	G5ECP9	vab-10	Plectin
HSP7A_CAEEL^‡^	P09446	hsp-1	Heat shock cognate 71 kDa protein
KARG2_CAEEL^‡^	Q27535	ZC434.8	Creatine kinase B-type

^x^*Minor components identified in both amyloid plaques and NFTs in Ayyadevara et al. (2016a).*

^†^*Minor components identified in amyloid plaques in Liao et al. (2004).*

^‡^*Minor components identified in NFTs in Wang et al. (2005).*

^∗^*Minor components identified as late-aggregating proteins in both day 10 and day 14.*

^+^*Minor component identified as early-aggregating protein with a high increase in aggregation at both day 10 and day 14*.

Consequently, this quantitative proteomic analysis of the *C. elegans* aggregating proteome at different ages brings insight into which minor components of pathological protein aggregates could directly seed disease-associated aggregation.

### Aged Mouse Brains Contain Protein Aggregates that Seed Aβ Aggregation *In Vitro*

**FIGURE 4:**
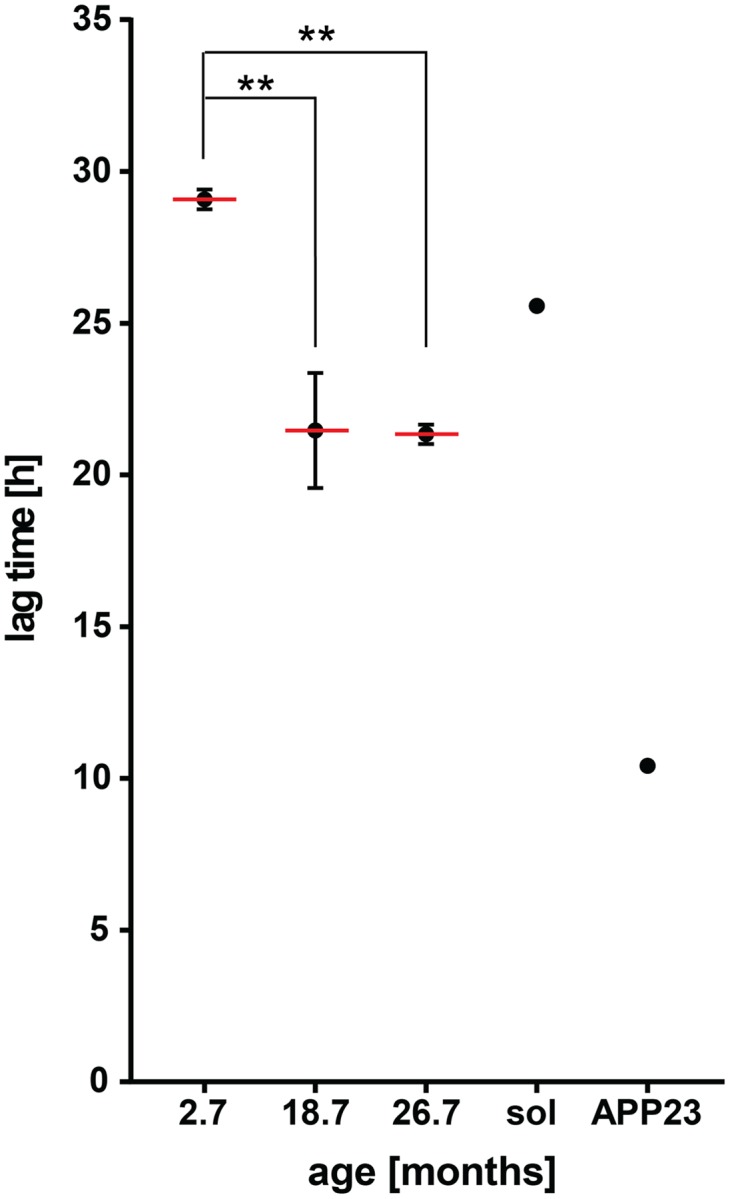
**Insoluble protein extracts from aged wild-type mouse brains seed Aβ aggregation *in vitro***. Lag times measured for Aβ aggregation in the presence of 0.001 μg detergent-insoluble protein extracts from wild-type mouse brains at different ages. Mean values (red lines) with respective SEM of three biological replicates (each including five technical replicates) from on average 2.7, 18.7, and 26.7 month-old mouse brain insoluble extracts. Lag time of an 18 month-old mouse brain soluble extract (sol, negative control) and a 20 month-old mouse brain insoluble extract from an APP23 transgenic mouse (APP23, positive control). One-way ANOVA: 2.7 months vs. 18.7 months ^∗∗^*p* = 0.0073, 2.7 months vs. 26.7 months ^∗∗^*p* = 0.0067.

To confirm that insoluble proteins present in mammals also have the potential to seed Aβ aggregation, we performed the same *in vitro* assay with homogenates of wild-type mouse brains. Of note, previous studies did not detect seeding activity in aged wild-type mouse brains (Nagarathinam et al., 2013; Fritschi et al., 2014). However, total brain homogenates were tested without any enrichment for insoluble proteins. Here, we measured detergent-insoluble protein extracts from young (2–3 months) and aged (18–20 months) mouse brains. Compared to young extracts, insoluble protein extracts from aged mouse brains led to a significant decrease of the lag time preceding Aβ aggregation (**Figure 4**, *p* = 0.0073, Supplementary Figure 4, *p* = 0.019). No further increase in seeding potential was detected when examining insoluble extracts from 25 to 28 month old mice (**Figure 4**, *p* = 0.0067 compared to 2–3 months). This is consistent with the similar seeding activity of extracts from late-middle aged and old *C. elegans*. For comparison, we included the insoluble extract from an APP23 transgenic mouse which contains Aβ seeds and the soluble extract from an aged wild-type mouse brain.

Collectively, these results demonstrate that cross-seeding of Aβ aggregation by age-dependent protein aggregation is conserved from *C. elegans* to mammals.

## Discussion

The discovery of widespread protein aggregation with age has raised the question whether these assemblies influence disease-related protein aggregation. Here, we demonstrate that a direct interaction between insoluble proteins from aged *C. elegans* or aged mouse brains initiates Aβ aggregation *in vitro*. These results could have important implications for our understanding of the initial pathogenesis steps in AD, particular in late-onset cases.

A recent study revealed that wild-type spinal cord homogenates injected into transgenic mice expressing α-synuclein with the A53T mutation induced early α-synuclein pathology (Sacino et al., 2016). As cortical homogenates from wild-type mice had no effect, the authors speculate that higher levels of myelinated white matter in the spinal cord could be responsible. A myelin component is unlikely to be the seeding agent in the data presented here. First, we performed a very stringent procedure to isolate highly insoluble proteins while removing the lipids using sucrose flotation and then high concentrations of strong detergents including SDS. Second, we used brain homogenates without spinal cord, brain stem, and cerebellum. Third, the effect observed is age-dependent and therefore unlikely to be due to the basic composition of the brain tissue used. Fourth, numbers of oligodendrocytes decrease with age (Pelvig et al., 2008). Therefore, it is reasonable to conclude that the induction of Aβ seeding in the present study is due to highly insoluble proteins rather than a lipid contaminant. In addition, we note that our findings cannot be explained by age-related changes in endogenous Aβ (Mahler et al., 2015) as *C. elegans* lack homologs of BACE and no Aβ-like peptides have been detected.

Previous *in vitro* studies have shown cross-seeding between Aβ and other disease-associated aggregating proteins including α-synuclein, PrP^sc^, hIAPP, tau and TDP-43 (Morales et al., 2013; Fang et al., 2014; Vasconcelos et al., 2016). Interestingly, acetylcholinesterase, a minor component in Aβ plaques, was also shown to accelerate Aβ aggregation *in vitro* (Inestrosa et al., 1996). This raised the possibility that minor components in disease-associated aggregates could also play a role in seeding the main component. Recently, this hypothesis has gained support with the discovery that these minor components are significantly over-represented in the age-dependent insoluble proteome (David et al., 2010). Therefore, a large number of minor components are themselves aggregation-prone. Our present data support the notion that misfolding and aggregation of minor components during age constitute heterologous seeding events for disease-associated aggregation. A recent study examined the expression levels of minor components found in plaques and NFTs in healthy brains (Freer et al., 2016). Interestingly, the regions known to be the first affected by NFT pathology in AD displayed higher levels of these minor aggregate components compared to non-affected regions. Of the 16 minor components identified as late- or early-aggregating proteins in our analysis, 7 out of 10 evaluated in the study by Freer et al. (2016) followed this pattern. Therefore, it is possible that higher levels of age-dependent seeds accumulate in these regions and induce tissue-specific vulnerability to disease protein aggregation in AD. Overall, it will be important to determine which proteins and in which locations have seeding activity.

Our proteomic timeline analysis highlights several minor plaque or NFT components that should be prime candidates as initiators of Aβ or tau seeding events to be investigated in future studies. One of these candidates, 14-3-3, has already been confirmed as a heterologous seed for tau aggregation (Li and Paudel, 2016). Importantly, we observed that 14-3-3 overexpression accelerated Aβ toxicity in a *C. elegans* model for Aβ aggregation. As some seeding proteins may not be sufficiently abundant to be detected in disease aggregates, it will be relevant to investigate the other late-aggregating proteins as well as early-aggregating proteins that continue to become more insoluble with age. In particular, proteins associated with the eight pathways highlighted by the IPA analysis should be promising candidates. Furthermore, it is possible that post-translational modifications occurring with age influence the seeding ability of these proteins. A number of post-translational modifications have been identified in disease-associated aggregates, and a recent study detected carbonylation in the mouse age-dependent insoluble proteome (Tanase et al., 2016). Another possibility is that changes in the structure of insoluble proteins could occur with age and explain why they become effective seeds.

A pressing aspect to be addressed in future studies is to determine the seeding activity of age-dependent protein aggregation *in vivo*. This could be done by using established mouse models for Aβ seeding, in which past experiments showed that intracerebral injection of Aβ-rich brain extracts in young APP-transgenic mice induced Aβ plaque formation prior to the appearance of endogenous Aβ deposits (Meyer-Luehmann et al., 2006). Of note, it is unclear whether this *in vivo* model would be sufficiently sensitive to detect heterologous seeding by the age-dependent insoluble proteome within the time window preceding the emergence of endogenous plaques and cerebral amyloid-β angiopathy. Indeed, our *in vitro* data showed a reproducible but relatively low seeding activity compared to Aβ seeds and therefore successful *in vivo* seeding studies may require repeated injections of insoluble extracts and/or very long incubation times.

Together, the present findings emphasize the need to understand better why protein insolubility is prevalent in older age and how the cellular quality-control systems fail to prevent it. Abrogating the formation of heterologous seeds could significantly reduce disease-associated seeding events and delay the onset of AD.

## Author Contributions

NG, FB, DD: conceived experiments, NG, AB, KWL, FB, DD: performed experiments; NG, CH, PvN, FB: performed data analysis; NG, KWL, FB, DD: wrote manuscript; AS, MF: revised manuscript; MF: contributed essential reagents; AS, FB, DD: supervised project.

## Conflict of Interest Statement

The authors declare that the research was conducted in the absence of any commercial or financial relationships that could be construed as a potential conflict of interest. The reviewer JH and handling Editor declared their shared affiliation, and the handling Editor states that the process nevertheless met the standards of a fair and objective review.

## Footnotes

1 http://www.socscistatistics.com/tests/fisher/default2.aspx

2 http://cildb.cgm.cnrs-gif.fr

3 http://www.ensembl.org/index.html

4 www.ingenuity.com
