# Supplementary material for: Age-Dependent Protein Aggregation Initiates Amyloid-β Aggregation
Source: Front Aging Neurosci. 2017 May 17;9:138. doi: 10.3389/fnagi.2017.00138 (PMC5434662; doi:10.3389/fnagi.2017.00138)
Supplement: Supplementary file 1 [file Data_Sheet_1.pdf]

Supplementary Material

Age-dependent protein aggregation initiates amyloid-β aggregation

Nicole Groh, Anika Bühler, Chaolie Huang, Ka Wan Li, Pim van Nierop, August B. Smit, Marcus Fändrich, Frank Baumann[#*], Della C. David[#*]

# Equal contribution

*** Correspondence:**
Della David, della.david@dzne.de or Frank Baumann, frank_baumann@gmx.de

Supplementary Figure 1

A

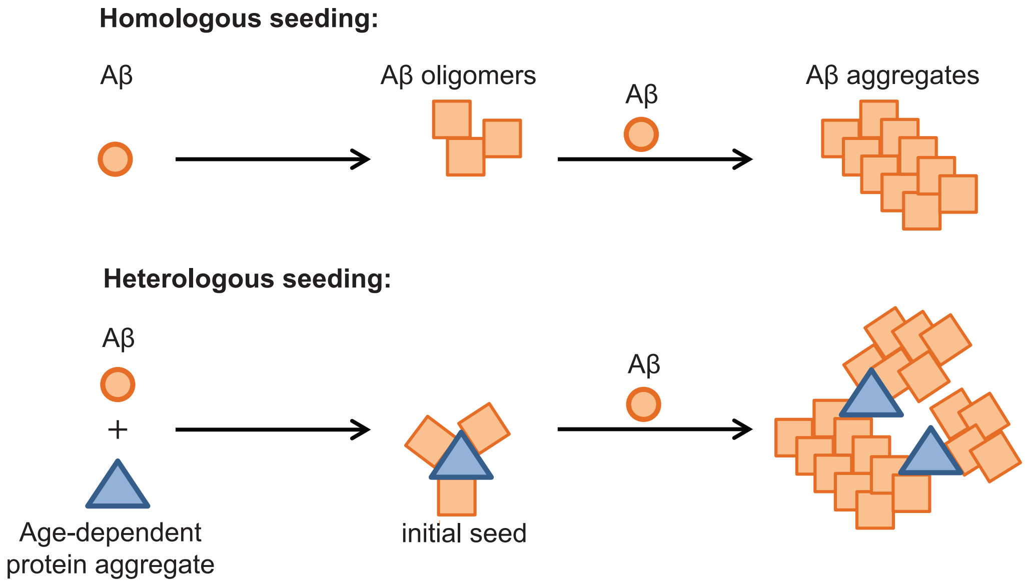

B

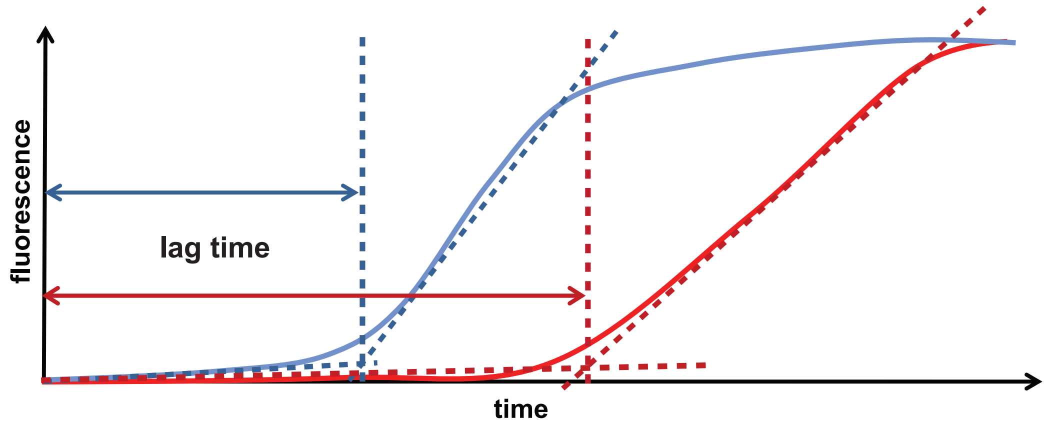

Supplementary Figure 1. Schematic representation of homologous and heterologous Aβ seeding and determination of lag times.
(A) Homologous seeding (upper part): Aβ monomers very slowly accumulate to metastable intermediate oligomers which act in a succeeding step as a seed for fast Aβ accumulation. Initial seed formation critically depends on exceeding a minimal local concentration of Aβ. This process leads to stable Aβ aggregate formation (fibril formation) which can break and serve as new seeds for further Aβ aggregation.
Heterologous seeding (lower part): Hypothetically during aging, Aβ monomers and age-dependent protein aggregates accumulate and build together an initial seed which leads to a faster accumulation of Aβ aggregates bypassing the slow buildup of homologous seeds from monomeric Aβ. This might be even more important when Aβ concentration never reaches the minimal local concentration for homologous seed formation.
(B) During the FRANK assay (Fibrillisation of Recombinant Aβ Nucleation Kinetic) protein extracts are measured in the presence of Thioflavin T (ThT) and recombinant Aβ1-40. Aβ fibrils, which are detected by the incorporation of ThT, are formed after initial fibril seeds develop. After this the ThT fluorescence increases rapidly until a maximum is reached (red line). The time that is needed to form initial seeds is called the lag time. Detergent-insoluble protein extracts of aged *C. elegans* or aged wildtype mouse brains reduce the lag time by initiating and accelerating Aβ aggregation (blue line).

Supplementary Figure 2

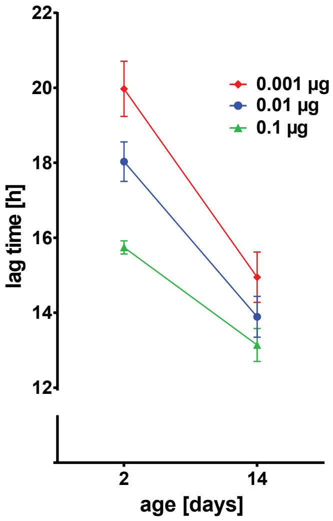

Supplementary Figure 2. Minute amounts of age-dependent aggregates seed Aβ aggregation. Lag times measured for Aβ aggregation in the presence of *C. elegans* insoluble protein extracts (day 2 and day 14). Three different amounts of insoluble protein extracts were evaluated: 0.1 µg, 0.01 µg and 0.001 µg. Mean values with respective SEM are represented (each including a minimum of five technical replicates). Two-way ANOVA day 2 vs. day 14: 0.1 µg $p=0.0084$, 0.01 µg $p<0.0001$, 0.001 µg $p<0.0001$.

Supplementary Figure 3

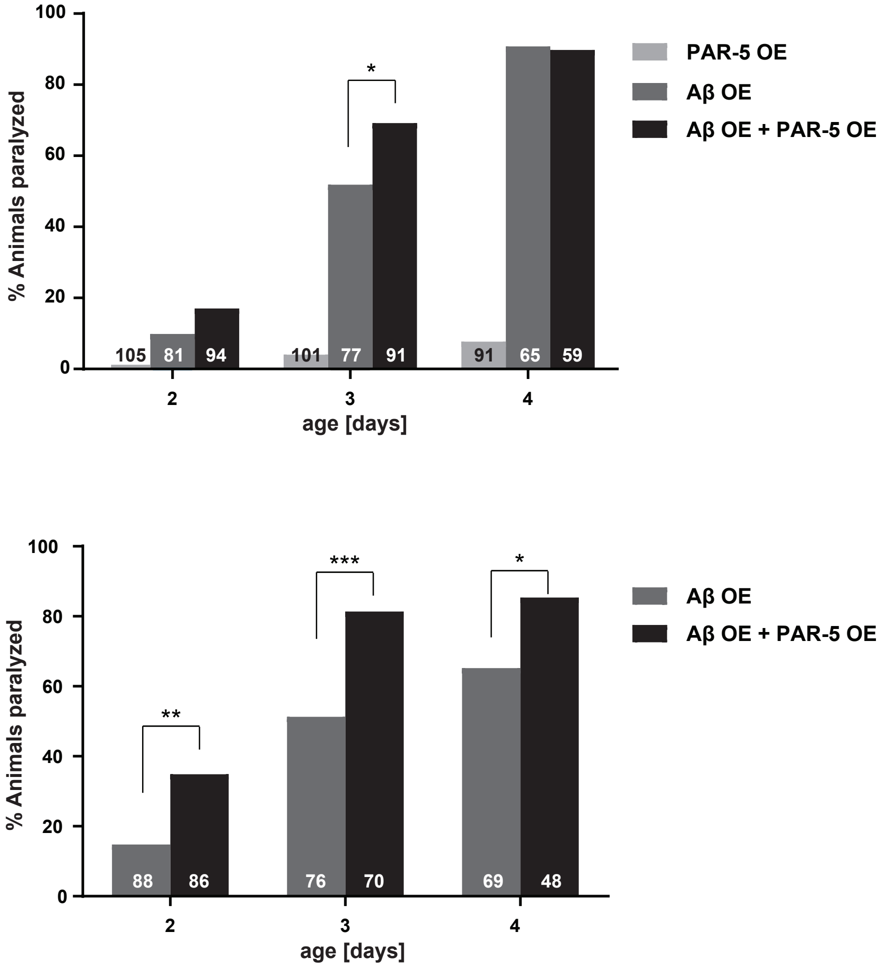

Supplementary Figure 3. Paralysis levels of worms overexpressing PAR-5, Aβ or both (double trangenic). Second and third repeat related to Figure 3. Shown are the percentages of worms paralyzed at different days. Numbers in the bars represent the total numbers of worms analyzed.
Fisher's exact test Aβ OE vs. Aβ OE + PAR-5 OE: Top: day 3 *$p=0.026$,
bottom: day 2 **$p=0.0026$, day 3 ***$p=0.0002$, day 4 *$p=0.019$.

Supplementary Figure 4

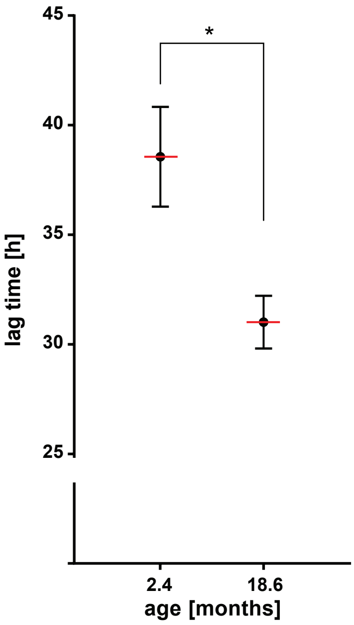

Supplementary Figure 4. Insoluble protein extracts from aged wildtype mouse brains seed Aβ aggregation *in vitro*. Lag times measured for Aβ aggregation in the presence of 0.001 µg detergent-insoluble protein extracts from wildtype mouse brains at different ages. Mean values (red lines) with respective SEM of five biological replicates (each including a minimum of 5 technical replicates) from on average 2.4 month and 18.6 month-old mouse brain insoluble extracts.
Two-tailed p value: 2.4 months vs. 18.6 months *p=0.019.

Supplementary Table 1. Mice used for FRANK Assay.

Number	Sex	Age (months)
7519	male	2
8418	female	2
8419	female	2
8628	male	3
8629	male	3
8916	female	2
8036	female	20
7830	male	18
7831	male	18
524	female	18
503	female	19
1736	female	20
7755	female	25
7718	male	27
7601	female	28
11523 (APP23)	male	20

Supplementary Table 2. Amount of worms grown in liquid cultures for different days for each biological replicate.

Replicate	Day 2	Day 6	Day 10	Day 14
1	100,000	100,000	150,000	200,000
2	150,000	150,000	200,000	350,000
3	250,000	150,000	175,000	300,000
4	170,000	150,000	150,000	225,000

Supplementary Table 3. Insoluble proteins from different ages of *C. elegans*.
Table is uploaded as Data Sheet 2.
First tab: 845 proteins were identified by mass spectrometry in two biological replicates. Proteins highly prone to aggregate with age (in the top 25th percentile) are marked in grey.
Second tab: Proteins quantified in both replicates as “early-aggregating” proteins (Top 25th percentile day 6-day 2) and as “late-aggregating” proteins (Top 25th percentile day10-day 6 and day14-day 6). Early-aggregating proteins in the highest aggregating fraction at day 10 and/or day 14 are marked in green and late-aggregating proteins highly prone to aggregate with age at both day 10 and day 14 are marked in blue.

Supplementary Table 4. Comparison of actual and expected numbers of proteins in the top 25th percentile of two biological replicates.

Proteins ranked in (two replicates):	actual	expected#	Chi-Square Test*
Top 25th percentile of day 6	133	52.8	4.38E-30
Not in top 25th percentile of day 6	712	792.2	
Top 25th percentile of day 10	88	52.8	5.71E-07
Not in top 25th percentile of day 10	757	792.2	
Top 25th percentile of day 14	137	52.8	5.45E-33
Not in top 25th percentile of day 14	708	792.2	
Top 25th percentile of day 6 and day 10	23	3.3	1.71E-27
Not in top 25th percentile of day 6 and day 10	822	841.7	
Top 25th percentile of day 6 and day 14	30	3.3	4.49E-49
Not in top 25th percentile of day 6 and day 14	815	841.7	
Top 25th percentile of day 10 and day 14	42	3.3	4.59E-101
Not in top 25th percentile of day 10 and day 14	803	841.7	

# Calculation: the expected probability of proteins to be ranked in the top 25th percentile of two replicates is 1/16. In addition the expected probability of these proteins to be ranked in the top 25th percentile of two different days is 1/256.

* Chi-Square Test has been performed with Excel formula CHITEST.

Supplementary Table 5. Ingenuity Pathway Analysis.
Table is uploaded as Data Sheet 3.
Ingenuity Canonical Pathways identified in the set of late- or early-aggregating proteins and their association with minor components found in AD pathological aggregates (highlighted in grey, –log(p-value) > 2). –log(p-value) and proteins included in the pathways are shown.
* late-aggregating proteins including early-aggregating proteins that continue to aggregate more at day 10 and day 14.
